# SLC34A3 Intronic Deletion in a New Kindred with Hereditary Hypophosphatemic Rickets with Hypercalciuria

**DOI:** 10.4274/jcrpe.601

**Published:** 2012-06-09

**Authors:** Shirin Hasani-Ranjbar, Mahsa M. Amoli, Azadeh Ebrahim-Habibi, Ehsan Dehghan, Akbar Soltani, Parvin Amiri, Bagher Larijani

**Affiliations:** 1 Tehran University of Medical Sciences, Endocrinology and Metabolism Research Institute, Tehran, Iran; +9821 88220037+9821 88220052shirinhasanir@yahoo.com

**Keywords:** Hypophosphatemia, hypercalciuria, hereditary hypophosphatemic rickets with hypercalciuria, nephrocalcinosis, SLC34A3 gene

## Abstract

**Objective:** Hereditary hypophosphatemic rickets with hypercalciuria (HHRH) is an autosomal recessive form of hypophosphatemia with hyperphosphaturia, hypercalciuria, and hypercalcemia. In two reports on six affected kindreds with HHRH, the disease was mapped to chromosome 9q34, which contains the SLC34A3 gene that encodes the renal type 2c sodium-phosphate cotransporter. Our objective was to define the clinical course of these cases in a family with HHRH and to screen for SLC34A3 gene in order to determine whether these mutations are responsible for HHRH.

**Methods: **After clinical and biochemical evaluations, the entire SLC34A3 gene was screened using PCR amplification followed by direct sequencing technique. In this paper, we describe a new kindred with HHRH and a case of progressive and complicated HHRH presenting at age 27 years.

**Results:** We found 101-bp deletion in intron 9 of the SLC34A3 gene. The index patient was homozygous for this mutation which has been previously reported in a Caucasian population. This is the first report for presence of SLC34A3 intron 9 deletion in an Iranian population.

**Conclusions:** These data showed that HHRH can be easily missed or underdiagnosed. Genetic evaluation of patients with familial hypercalciuria, hypophosphatemia and nephrolithiasis is needed for further information on the prevalence and management of this rare disorder.

**Conflict of interest:**None declared.

## INTRODUCTION

Phosphorus is a mineral essential for numerous cellular functions and is an important constituent of bones. Hypophosphatemia in children leads to rickets resulting in poor growth and frequently in skeletal deformities. Among the various causes of low serum phosphorus are inherited disorders associated with increased urinary excretion of phosphate, which include X-linked hypophosphatemia (XLH), autosomal recessive hypophosphatemic rickets (ARHR), autosomal dominant hypophosphatemic rickets (ADHR), and hereditary hypophosphatemic rickets with hypercalciuria (HHRH) (1). Clinical and biological characteristics of hypophosphatemia, as well as its management, depend on the specific etiology (2). HHRH is an autosomal recessive form of hypophosphatemia that has been recently described with hyperphosphaturia, hypercalciuria, hypercalcemia, decreased serum parathormone (PTH) levels and increased serum alkaline phosphatase (ALP) activity (3,4,5,6). In addition to cases from a Bedouin tribe and a kindred of Jewish Yemenite origin, a few sporadic cases of this disease have also been reported (5,6,7,8,9,10). In two reports on six kindreds affected by HHRH, the disease was mapped to chromosome 9q34, which contains the SLC34A3 gene that encodes the renal type 2c sodium-phosphate cotransporter (10). We describe here a new kindred with HHRH. The index subject was a man with progressive and complicated HHRH, who presented to our clinic at age 27 years, with no prior treatment.

## METHODS

**Patients and data collection**

**Figure 1:**
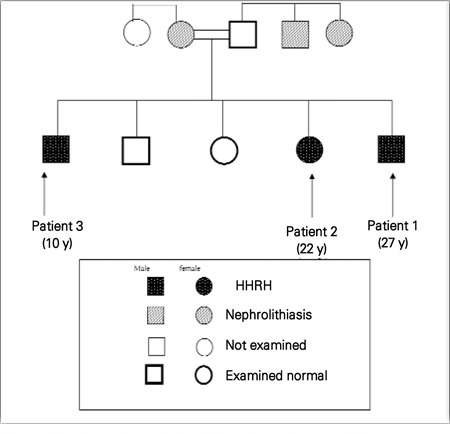
Genetic relationship of patients with hereditaryhypophosphatemic rickets with hypercalciuria (HHRH) and 3 subjectswith hypercalciuria and renal stones

The family (Figure 1) was evaluated for past medical history. The patient’s physical examination and biochemical assessment were performed in the endocrine unit of the Shariati Hospital, Tehran University of Medical Sciences. Blood samples were taken after a 12-hour overnight fast. In addition, urine was collected for 24h to measure calcium, creatinine and phosphorus. Tubular reabsorption of phosphate (TRP) was calculated using the following formula: 1 - (urine phosphorus X serum creatinine/serum phosphorus X urine creatinine). Then TMP/GFR (tubular maximum reabsorption threshold of phosphate per glomerular filtration rate) was calculated using a nomogram developed by Walton and Bijvoet (1,10). Skeletal X-rays were taken, and renal ultrasonography was performed. Genetic analysis and protein modeling were also conducted.

**Genetic analysis**

Genomic DNA was isolated from peripheral blood leukocytes using the salting-out method. The entire SLC34A3 gene which spans approximately 5 kb of genomic DNA was amplified, followed by direct sequencing according to the assay described by Bergwitz et al (11).

**Protein modeling**

The sequence of sodium-phosphate cotransporter type 2c (gi|25014088) was retrieved from NCBI protein (http://www.ncbi.nlm.nih.gov/protein). Secondary structure prediction of the protein was performed with the use of the HMMTOP server (www.enzim.hu/hmmtop) (12). The obtained predicted structure was schematically drawn with the help of TOPO2 (Johns SJ, TOPO2,Transmembrane protein display software, http://www.sacs.ucsf.edu/TOPO2/). Translation of nucleotide to protein sequence was performed with the use of EXPASY translate tool (http://web.expasy.org/translate/).

## RESULTS

**Subjects**

Patient 1 (Index patient, Figure 1). A 27-year-old man, whose parents were distant relatives, was referred to our clinic for evaluation of skeletal deformities, bone pain and nephrocalcinosis. He presented with leg deformities and a history of progressive departure from normal growth starting at age 10 years. At age 12 years, he had undergone an operation because of bladder and kidney stones, and a second surgical intervention had been performed at age 25 years. The patient also had a history of early dental caries. By age 18 years, his physical activity had become very restricted. He had no drug addictions. He had received courses of antibiotic therapy for recurrent urinary tract infections.

At presentation, muscle weakness was not a prominent feature. The patient’s height was 110 cm (SD score: -9.0) and his weight was 50 kg. He displayed striking deformities in his chest and in his upper and lower limbs. Laboratory data revealed hypophosphatemia, elevated ALP activity and hypercalciuria. TMP/GFR was 1.4 mg/dL and 24-hour phosphate excretion was 1080 mg (Table 1). Urine culture was positive for Escherichia coli.

**Figure 2:**
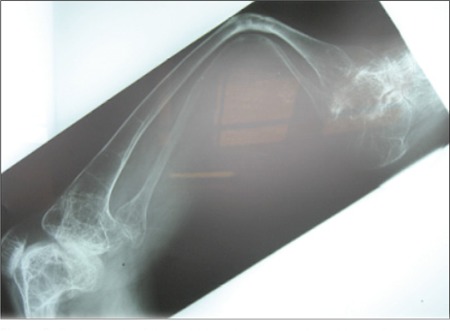
Radiograph of the right leg showing profound loss of bone withsevere bending of the bone shafts and thin cortical layer

Radiography of the limbs revealed late complications of rickets (Figure 2). Renal ultrasonography showed that the right kidney size was 77?33 mm and the left kidney size was 111?50 mm. There were two renal stones in each kidney. No signs of hydronephrosis were noted.After evaluation, the patient was treated with neutral phosphate only. Two gram of elemental phosphorus per day was given orally in 3 divided doses. Bone pains disappeared within three weeks. Serum calcium decreased and reached a near-normal level. Serum phosphorus level (in fasting state, 2 hours after the first dose of neutral phosphate) increased toward normal. TMP/GFR remained unchanged.Patient 2 was a 22-year-old male (Figure 1) who had short stature (SD score:-4.5), skeletal deformities and nephrolithiasis. The results of laboratory tests are shown in Table 1.

Patient 3 (Figure 1) was a 10-year-old boy who also had bone deformities and renal calculi. He was diagnosed in another clinic and his laboratory data were not available. His family history was positive for nephrolithiasis in his mother, maternal uncle and aunt and they were all under treatment with thiazide.

**Genetic analysis**

**Figure 3:**
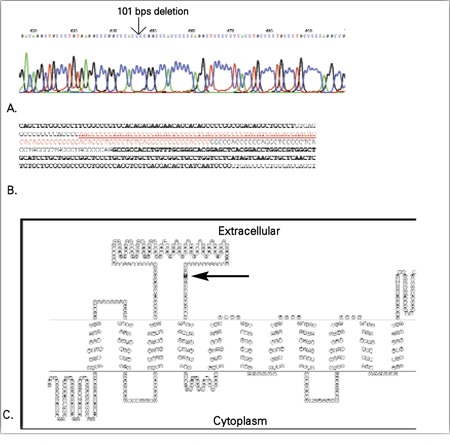
Mutation analysis of SLC34A3 gene and protein model. Thesegment of the chromatogram with arrow indicates the site of deletion inSCL34A3 gene in our patient

Mutation analysis revealed presence of 101-bp deletion in intron 9. The index patient was homozygous for this mutation. Figure 3 illustrates the presence of deletion in our patient.

**Protein modeling**

Deletion of the indicated segment in intron 9 (Figure 3B) has been experimentally shown to result in intron retention in the mRNA (13). The 22 amino acids that would result from this insertion have been found to have the following sequence: EARPTPAHPPGSPSPAPALPPG. Since no three-dimensional structure has been reported for this protein or closely related ones, a secondary structure prediction was performed in order to get an insight into the putative position of the added residues. The results obtained from the HMMTOP server were used, based on the fact that this algorithm was observed to predict a more correct topology for the similar sodium-phosphate cotransporter IIa (from rat) (14), compared with other existing algorithms. The hypothetical representation of sodium-phosphate cotransporter 2c topology has been drawn with the use of TOPO2, and contains 11 transmembrane segments (Figure 3C). The putative addition of amino acids would occur after C309, whose position is indicated in Figure 3C.

## DISCUSSION

It should be emphasized that the index patient (patient 1) manifested severe short stature and bowed legs, and the biochemical assessment revealed hypophosphatemia, phosphaturia and calciuria. As a matter of fact, hereditary forms of hypophosphatemic rickets that are associated with renal phosphate loss include X-linked and autosomal dominant disease as well as HHRH. The latter disease, the only form that can explain hypercalciuria, is very rare (1,2). The etiologies of early-onset nephrocalcinosis in consanguineous families include five major inherited recessive disorders, including HHRH (3). The development of nephrocalcinosis in X-linked hypophosphatemic rickets is probably related to intermittent episodes of hypercalcemia and hypercalciuria. These can result from an excessive calcitriol dose or from noncompliance with oral phosphate supplementation and is associated with the dose of phosphate received (4,15). On the other hand, in patients with HHRH due to hypophosphatemia, an appropriate elevation in the serum levels of 1,25-dihydroxyvitamin D leading to hypercalciuria, hypercalcemia and decreased serum PTH levels and increased serum ALP activity are noted (5,6). Elevated ALP is due to hypophosphatemia itself. These biochemical features were observed in two of our patients. Most HHRH patients presenting with rickets have short stature and increased renal phosphate clearance (low TMP/GFR) (1). In homozygous state (as in our index patient) or in compound heterozygoty, urinary loss of phosphorus is more significant, resulting in low serum phosphorus and rickets (16). However, milder forms may be underdiagnosed if the hypophosphatemia is mild; such patients present with hypercalciuria and nephrolithiasis, but no signs of bone disease are observed (5). These heterozygous patients have renal stones without rickets (16).

HHRH was initially described as a new syndrome in a large Bedouin tribe kindred (7,8). Up to now, only a few cases including a kindred of Jewish Yemenite origin as well as familial and sporadic cases from Europe, North America and Japan have been reported (7,8,9,10). We described here HHRH in an Iranian family. These family members (some with progressive HHRH) were diagnosed in another health facility as familial nephrolithiasis. They were also advised not to use calcium, phosphate and vitamin D supplementation. These findings lead us to think that HHRH is a condition that is underdiagnosed possibly due to its similarity to other hypophosphatemic syndromes or familial nephrolithiasis in its clinical, radiological and most biochemical parameters. Patients with the hypercalciuric form of hypophosphatemic rickets should be treated with phosphorus supplementation alone (1). The addition of active vitamin D metabolites could cause deterioration in the patient's condition (7). In skeletally immature patients, the goals of therapy are attainment of a normal longitudinal growth rate and achievement of normal serum ALP concentrations, rather than normalization of serum phosphate concentration. On the other hand, in skeletally mature patients, the goal of therapy is to prevent the symptoms of hypophosphatemia (7,17). We should also stress, as also noted in our patient and his family who had to undergo operation because of kidney stones, that kidney stones and refractory urinary tract infections are important complications of the disease.

HHRH is inherited in an autosomal recessive manner (18). The disorder is known to be caused by genetic mutations of the renal type 2c sodium-phosphate cotransporter. In two reports on six affected kindreds with HHRH, the disease was mapped to chromosome 9q34, which contains the SLC34A3 gene that encodes the renal type 2c sodium-phosphate cotransporter (10,13,19,20,21). However, the correlation between gene abnormalities and osteoblastic function in human HHRH osteoblasts is still unclear (9). Genetic analysis in our study showed a previously reported deletion in intron 9 in the SLC34A3 gene. This is the first report for presence of this mutation in an Iranian patient. It has been suggested that the presence of identical sequence repeats in this region might have endorsed misalignment during meiosis causing such a deletion. Large region deletions in small introns are not frequently reported in other conditions. As the resulting mRNA was observed to contain the truncated intron with 66 bp (13), a putative addition of 22 amino acids may occur in the potentially produced protein. In this case, the segment would be located in the second extracellular loop of the protein (Figure 3C), probably affecting the correct folding of the protein afterwards, resulting in a nonfunctional product. Even though this extra cellular loop is of a large size and should probably be able to accommodate for smaller insertions, in this case, addition of a 22-amino acid segment would result in too much change in the consecutive membrane segments. HHRH is an autosomal recessive disease and our patient in this study was homozygous for SLC34A3 gene mutation, therefore, this patient must have inherited the mutation from both parents for complete disease manifestations. However, the presence of nephrolithiasis in the family members might indicate some related clinical phenotype occurring in the presence of one mutated allele.

Our data has also shown that HHRH can be easily missed or underdiagnosed. Genetic evaluation of patients with familial hypercalciuria, hypophosphatemia and nephrolithiasis are needed for further information on the prevalence and management of this rare disorder.

**Acknowledgments**

We gratefully thank the patients and their family for consenting to the publication of this study.

## Figures and Tables

**Table 1:**
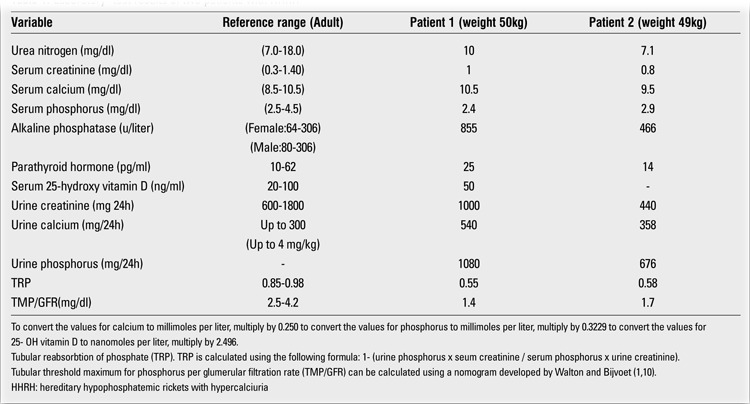
Laboratory- test results of two patients with HHRH
